# Transport and Mobility Needs for an Ageing Society from a Policy Perspective: Review and Implications

**DOI:** 10.3390/ijerph182211802

**Published:** 2021-11-10

**Authors:** Dong Lin, Jianqiang Cui

**Affiliations:** 1 School of Engineering, University of Aberdeen, Aberdeen AB24 3UE, UK; dong.lin@abdn.ac.uk; 2 School of Engineering and Built Environment, Griffith University, Brisbane, QLD 4000, Australia

**Keywords:** old people, policy, mobility, legislative and institutional approach, ageing

## Abstract

The world population is ageing, and many countries in the world are confronted by significant challenges in adapting their health and social systems to meet the requirements of this demographic change. It is well-accepted that mobility is often closely linked to a person’s independence, wellbeing and quality of life. Interest has increased across the world with regard to how the mobility needs of an ageing society can be addressed, since an ageing population is confronting nations across the globe and poses long-term challenges on a nation’s many aspects, including transport. Questions of how future policies can better respond to the mobility needs of an ageing population and how these policies can be delivered have emerged as major concerns for transport planners, operators and decision-makers. This review explores the way in which implemented policies have addressed the mobility needs of an ageing society, focusing on the provision of accessible, safe and affordable transport. More specifically, the paper reviews legislative and institutional approaches of addressing the mobility needs of an ageing population. The paper discusses the outcomes of these approaches and the remaining challenges in this policy area that relate to elderly people’s mobility and provides future research directions. Based on the discussion, clear conclusions about the effectiveness of particular policies are difficult to make. Alternative approaches (e.g., technological innovations) need to be considered in addressing older people’s mobility needs.

## 1. Introduction

People are living longer worldwide. According to the World Health Organization (2018), for the first time ever, most people can expect to reach their 60s [1]. The number of older people in the world has increased dramatically. People aged 65 years and over accounted for 4.97 percent, 6.16 percent and 8.87 percent of the total population in the world in 1960, 1990 and 2018 respectively, showing an increasing trend in the past few decades [2]. The proportion of older people over 60 years in the world will almost double from 2015 (12 percent of the population) to 2050 (22 percent of the population) [1]. One-sixth of the world’s population will be over 65 years old by 2050 [3]. The number of elderly people is believed to be an essential measure of ageing because it significantly impacts health and social care provisions, housing demand, and long-term care [4]. Globally, there was an increase of more than 170 million people aged 65 years and over in the decade from 2008 to 2018 [2]. Developed economies have older demographic profiles; for example, more than one-fifth of the population in Europe and North America were aged 60 or over in 2017, and the figure was predicted to be 35 percent in Europe and 28 percent in North America respectively in 2050 [5]. The considerable economic and social consequences of ageing in developed countries in Europe and North America are well-known. In comparison, the slower adoption of ageing policies in developing countries remains a concern, as these countries have an even faster pace of ageing than developed countries. For example, it would take 26 years for the proportion of the population aged 65 and over to increase from 7 percent to 14 percent in China and 26 years for the same to happen in Brazil, while much longer time periods have been historically recorded in developed countries—115 years in France, 85 years in Sweden, and 65 years in Canada [6].

People now have a longer life expectancy and varied lifestyles and are healthier in later life. Ageing populations and the increasing burden of dependency associated with an ageing demographic profile have significant economic and social consequences. Mobility, closely related to the basic human need for physical movement (and therefore independence) of the elderly in their everyday lives, can promote healthy ageing [7]. Mobility is closely associated with quality of life and wellbeing in later life [8]. The future members of an ‘older population’ have lived in a society with modern mobility, characterised by automobile transport and long-distance travel for leisure in many developed economies. They have benefited from the welfare and healthcare systems that ensure that they are healthier and live active lives with varied leisure activities, compared with previous older generations. Older adults nowadays can be expected to remain independent and active and use transport systems frequently until they are 80 years old [9]. On the other hand, developing countries are confronted with ageing demographic profiles while the levels of per capita income are considerably lower than for developed economies [4]. Rapidly ageing populations, alongside a slower adoption of ageing as an important public policy in these countries, present an urgent need for the development of appropriate policies to facilitate the elderly’s independent travel.

It has been highlighted that traditional transport policy for addressing mobility needs of the elderly tends to focus on the needs of the less able-bodied elderly. For instance, various measures have been implemented in the United Kingdom (UK) to cope with the effects of age-associated disabilities that restrict mobility. Accessibility to public service vehicles for elderly people with disabilities was guaranteed through the *Disability Discrimination Act 1995* [10]. However, many researchers (such as [11,12]) recognise that the focus on the less able-bodied elderly in traditional transport policy requires a revolution.

The transport needs of older people are not homogeneous. Their needs are varied and can be affected by a considerable number of influencing factors [13]. Lifestyle (such as whether people are working or retired, and housing styles) and socio-demographic characteristics (such as age, gender, income, driver licence possession, and household size and structure) can significantly impact on the transport needs of older people [14]. In this car-dependent era, when travel without a car can often be difficult or inconvenient, particularly for vulnerable groups of people including the elderly, transport policy should consider the use of other transport modes, e.g., public and active transport. In this way the mobility needs of the elderly who are unable or unwilling to use cars can be addressed. The implementation of appropriate transport policy would reduce transport related social exclusion among older adults.

There is a need for a commitment regarding three major policy areas: the provision of mobility options, legislative and institutional approaches, and building accessible mobility environments [12]. Among the abovementioned policy areas, providing mobility options and ensuring accessible mobility environments have been intensively researched (e.g., [15,16,17,18,19,20,21,22,23,24,25]). While the provision of mobility options and building accessible mobility environments have been well-investigated and documented in the transport policy area, the role of legislative and institutional approaches in transport provision is not clear. This review, focusing on legislative and institutional approaches in transport provision, addresses this gap. Legislative and institutional approaches are important components of transport policy development that have been considered in addressing transport provision for elderly population by directly impacting on older people’s transport preferences or through other influencing factors such as lifestyle and financial incentives. Governments play an important role in providing transport options for essential social services for the elderly, accounting for their specific needs. Therefore, investigating current legislative and institutional approaches for addressing the elderly’s mobility needs to obtain a better understanding of developing policies for promoting equity and social inclusion has great significance in research and for practical purposes.

Most studies of policies for addressing older people’s mobility needs are limited in scale and are usually restricted to individual case studies of a particular policy in a specific region (e.g., [26,27,28,29,30,31]). Therefore, little is known about policies implemented worldwide and about their outcomes in different contexts across the globe. Developing an understanding about the way in which legislative and institutional approaches, implemented for addressing older people’s mobility needs, are appropriate, and the contexts in which they are appropriate proves challenging. This article aims to address such questions by exploring empirical evidence regarding policies implemented globally in response to the mobility needs of an ageing society, by paying special attention to legislative and institutional approaches. This review provides researchers and practitioners with an up-to-date understanding of legislative and institutional approaches in addressing transport provision for an ageing society. The main research questions are: (1) What policies have been implemented for addressing the mobility needs of ageing populations, particularly with regard to legislative and institutional options; (2) How have these policies been implemented; and (3) What were the outcomes and issues? The research objectives are to map out the existing knowledge and to synthesise empirical evidence on the role of legislative and institutional approaches to transport provision, and issues that arise from these approaches, focusing on scholarly sources.

The review is significant and valuable because it provides a review of the policies on transport provision from a new perspective, including the legislative and institutional approaches. In addition, based on existing knowledge about transport policy for an ageing society, this review develops a framework for exploring transport provision for an ageing society from legislative and institutional perspectives, to help guide the planning and implementation of transport policies for addressing older people’s mobility needs by authorities across the globe.

## 2. Methodology

**Figure 1:**
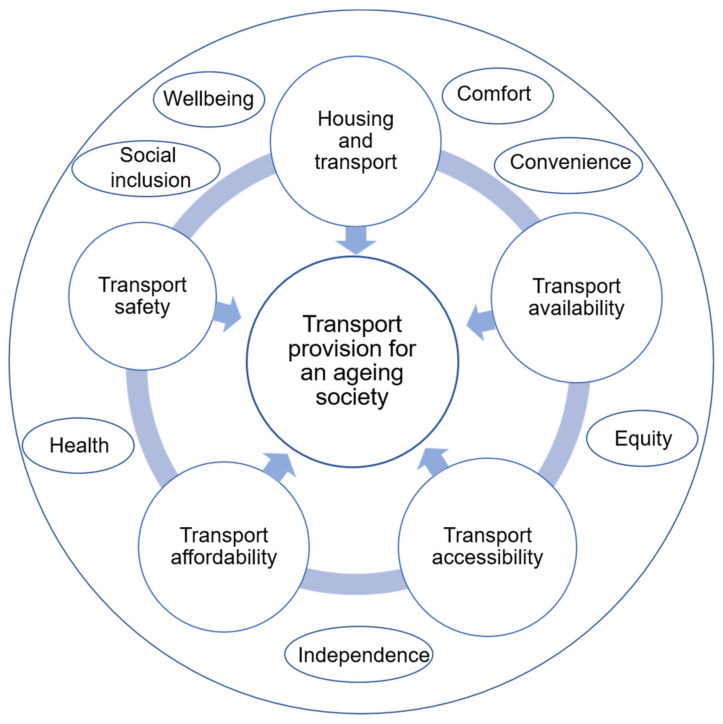
A framework for the review of transport and mobility needs for an ageing society from a policy perspective.

A considerable number of studies examined the relationship between older people’s mobility and travel behaviour and the built environment, and the key objectives of transport planning and policies for older people. Housing, and the neighbourhood environment, both of which impact on travel behaviour are key policy areas to focus on [32,33]. The provision of accessible and affordable transport is also an important objective in planning for the mobility of older people [34]. Research has indicated that the availability, accessibility and affordability of transport play an important role in affecting older people’s travel preferences and addressing their mobility needs [35,36,37]. The availability, accessibility and affordability of transport have consequences for equity, comfort, convenience, health, independence, wellbeing and social inclusion all of which are important considerations in transport provision [38,39,40,41,42]. The framework of the review is shown in Figure 1.

Based on the review framework, this review draws on the authors’ expertise in this area, with information and data sourced from journal publications via Google Scholar and ScienceDirect searches, using search terms including ‘transport policy’, ‘housing’, ‘affordability’, ‘availability’, ‘accessibility’, ‘safety’, ‘travel behaviour’, ‘mobility’, ‘regulation’, ‘subsidy’, ‘public transport’, ‘driver license’, ‘active transport’, ‘the elderly’, ‘older people’, ‘senior’, and ‘ageing’. We focused on the affordability, availability, accessibility and safety aspects of transport provision for the elderly. Private cars, public transport and active transport were considered. This was followed by a second-round inspection of the reference lists of the identified papers to increase the number of publications available for the analysis. Reference made to these papers in the review depends on the relevance and value of each publication to the research questions. Although a considerable number of publications are identified through database searching, many have been excluded from the review according to the inclusion and exclusion criteria listed in Table 1. A total of 46 publications are included in this review. Existing studies focus on developed countries, and comparatively fewer studies have been conducted in developing countries. This review presents research findings for many countries, particularly for Australia, Canada, China, Denmark, Germany, Israel, Japan, Korea, The Netherlands, Norway, Sweden, the United States (US), and the UK. The following sections discuss the review findings with regard to housing and transport, and transport availability, accessibility, affordability and safety. It is worth noting that review findings in the existing literature regarding transport availability and accessibility are not easy to differentiate between. To better display the research findings of the existing literature, we have combined the two in the same section.

## 3. Housing and Transport

Patterns of land use and related housing arrangements provide the contours of older adults’ daily lives. Considerable research has supported ‘ageing in place’ as the preference and trend for housing provision for older adults in Hong Kong, China [43,44], Korea [45], Australia [19] and the US [46]. In Hong Kong, ‘ageing in place’ has been a leading principle of the services provided to older people by the Government of the Hong Kong Special Administrative Region (SAR), while government residential care services or nursing homes are the final options considered for older people. The government of the Hong Kong SAR has invested heavily in a public housing program, alongside community care policies, to promote ageing in place for older people [43]. It has been noted that for those ageing in place, a large proportion of older people live in suburban or rural areas where reliable public transport is not provided, and therefore all types of travel largely rely on car transport. Affordability is one of the main considerations. In the US, popular retirement destinations are built around car use as a result. Even they are located in transit-rich regions with relatively affordable land, the community built for older adults established in suburban settings usually fails to provide sufficient facilities, such as sidewalks and lighting [46]. Kim’s (2011) research on older people in the US suggested that older people living in suburban communities suffered from considerable transport deficiency. The provision of activity clusters containing commercial and social service facilities within walking distance reduced non-driving seniors’ transport deficiency [47]. 

The trend towards urban–rural migration of older adults would provide guidance for policy-making on housing and transport provision. The Korean government anticipated that the elderly would move from urban to rural areas when retired, and this has been regarded as a contributing factor in the economic growth of rural areas over the coming years. Accordingly, it was assumed by the Korean government that it is vital to provide health services and sufficient housing in rural areas for the elderly. Surprisingly, Kim and Han (2014) revealed that over the past ten years, the elderly had relocated to high-density areas. The very elderly cohort, over 70 years old, tended to move to areas of high density where health services and living amenities can be accessed easily [45]. The research findings are different from the Korean government’s anticipation on the trend of urban–rural migration of older adults, which indicated the requirement of detailed investigations prior to the introduction of housing and transport policies.

The elderly constitute part of the low-income group that receives housing subsidies. In Canada, low-income seniors can be in receipt of government-subsidised senior citizen apartments (SCA) projects of age-segregated congregate facilities. Residents of SCAs may be directly subsidised or pay rent according to their incomes. Older people who have health conditions, family difficulties or financial hardship, may results in them moving to an SCA. Sylvestre and Smith’s (2009) research found that most of the older people moving to SCAs came from their home neighbourhoods or nearby, so that one or more of their adult children were living within a short distance of their new homes after relocation. In this way, for assistance that was typically unavailable at SCAs (e.g., the provision of automobile transport), the adult children of the elderly living in SCAs were able to offer the assistance required. It was indicated that the elder’s desire to maintain spatial proximity with their adult children may discourage their considerations of SCA opportunities involving longer-distance relocation [48]. Older residents (aged 65 years and over) in British Columbia, Canada who spend over 30 percent of their household income on renting their residences are eligible for a Shelter Aid for Elderly Renters (SAFER) rental subsidy through BC Housing, a provincial Crown organization. Chudyk et al. (2015) investigated older adults in receipt of the rental subsidy and found that this group of low-income older adults relied on walking and public transport for their travel needs. With more attractions or ‘destinations of interests’ in their immediate neighbourhoods, the elderly appeared to be more willing to walk to places that were close-by instead of driving a car [17]. 

Transport considerations play an important role in the elderly’s housing selection. A survey of Hong Kong’s older people and their housing preferences revealed that transport convenience and proximity to markets are the most important factors selected by the majority of respondents in their housing decisions [43]. Accordingly, housing provision can have a significant impact on the location of older people in retirement and thus their travel behaviour and mobility needs. 

## 4. Transport Availability and Accessibility

The issue of transport availability has been proven to contribute to declining levels of mobility at older age [49]. According to Metz (2003), the private car is an ideal transport mode for the elderly, as it provides an autonomous, affordable, time-flexible and door-to-door transport option [10]. Since cars were found to dominate the travel modes of the elderly [16,50], research has highlighted the legislative and institutional approaches of supporting transport infrastructure and services for car transport. Driving license possession policies impact on older people’s travel behaviours significantly. To illustrate, in Denmark, Norway and Sweden, the growing number of driving license holders and the increased car availability would impact on the travel modes of the elderly, resulting in more seniors making their daily trips by driving, until they are 80–85 years old [51].

Many studies have indicated that losing a driver’s license can have negative effects on older adults with respect to health, mobility, safety, independence and quality of life [18,27,52]. Adult children worried about the responsibility they would undertake when their parents ceased driving [53]. A study examined the Sydney Household Travel Survey in 2002–2004 and revealed that the loss of a driver’s license can lead to social isolation if adequate flexible public transport and/or support mechanisms that enable car passengers are unavailable [54]. In fact, an increasing number of older adults are using cars as their main mode of transport [54], and thus an increase in the number of driving license holders among older adults can be expected. It indicates that in the future more older adults may use cars for their daily trips, up to the age of 80–85 years, and this would provide significant implications for licensing policy [51]. Furthermore, the gender difference between male and female older drivers is also worth noting. Swedish and Norwegian data have shown that among the age group of 80–84 years, 80 percent of males hold driving licenses, while the figure is only 28 percent for females [51].

It is known that most old people consider decreasing or ceasing driving at some stage, and this would cause a significant decline in mobility and an increase in physical and mental health risks if appropriate transport alternatives are not available. An Australian study of elderly people aged 75 years and older found little evidence of planning for and support in decision-making about ceasing driving, indicating that resources to assist the elderly, their carers and health professionals to plan for alternative transport options for elderly people’s transition from being a driver to non-driver are required [55]. Planning for the transport needs of seniors is required to be placed on future planning agendas by planning authorities. However, a US nation-wide survey of Metropolitan planning organizations (MPOs) on their understanding about where ageing was on their planning agenda as well as what projects were in their 2-year and long-range strategic plans revealed that the majority of the MPOs would not meet the likely needs of the ageing population within twenty years with regard to adequately funding infrastructure, vehicles and services. Surprisingly, the majority of the MPOs agreed that older adults in the near future would rely on their own vehicles, or their families’ or friends’ vehicles to meet their transport needs [46]. Despite the acknowledgement of the need for resources to support elderly people once they no longer drive, an interesting study in Australia investigating female drivers and former drivers aged 60 years and over found that current drivers were extremely interested in maintaining driving behaviour for as long a period as possible. They were highly concerned about ceasing driving and showed little evidence of self-regulation. In contrast, former drivers reported they were less negative about stopping driving and mostly were successful in the transition from being drivers to other transport mode users, with few negative mobility consequences [56]. This indicates that there may not be overwhelmingly negative viewpoints about the consequences for older drivers of stopping driving. The way in which to increase awareness and confidence for a successful transition from being drivers to other transport mode users, and to provide robust support to facilitate such a transition at the appropriate times require further investigation.

The provision of multiple transport options (particularly sustainable modes of transport in the future) is important to maintain the independent travel of the elderly [57]. Many researchers expressed their worries that alternative transport arrangements provided may not compensate for the loss of mobility due to the cessation of use of cars, after taking affordability, efficiency, convenience, comfort and enjoyment into consideration [18,27,47,58]. 

Considering that impairment generally increases with age, the accessibility of transport infrastructure and services for the elderly and the disabled has received much attention. Many developed countries (e.g., Australia, Canada, Sweden, the UK, and the US) have introduced legislation, regulation standards, or codes of practice to ensure accessible transport [59]. Developing countries are moving towards legislation that requires transport services to be made accessible, although the translation of accessibility policies into the provision of inclusive transport remains a major obstacle for many reasons, e.g., lack of monitoring and enforcement of compliance with existing accessibility legislation and inadequate resources for implementation [60]. An Australian study examined the equality issue related to accessible trams (low-floor trams) in Melbourne and found that 70 percent of the population with disabilities only have access to 22 percent of the accessible trams. Comparatively, 70 percent of the entire Melbourne population can access 40 percent of the trams [61]. Therefore, the provision of accessible tram services for disabled/older people is an equity concern. Budd and Ison [62] investigated disabled air passenger rights legislation that requires air transport operators to provide minimum service standards and levels of service provision to disabled travellers, based on an international survey of 47 countries. They reported a lack of global standards for disabled air passengers, and only 6 countries/regions among the 47 countries/regions had dedicated laws or regulations related to the rights of passengers with disabilities. A consequence of the inconsistency of the disabled air passenger rights legislation among different countries could be that the needs of passengers with disabilities are only met or partially met at some stages of a journey, depending on their travelling locations and time.

Sometimes, travelling by public transit proves to be inconvenient. Public transport is rarely available for some special occasions such as family gatherings or funerals. In addition, poor access to bus stops, long walking distance, inadequate facilities (e.g., shelters and seats), and burdens associated with the interchanges of public transport (e.g., obtaining interchange information, carrying luggage and finding the way) hinders the use of public transport for older people [18,26,63]. Many older adults lag behind younger people with regard to technology adoption (e.g., they often do not use computers or smart phones to check travel information, and do not know how to use vending machines for tickets) [58]. What is even worse, is that in some regions (e.g., the southern Swedish region of Scania), older people tend to move to the countryside after retirement where the quality of public transport service is even poorer compared to urban areas [64].

There is a great potential for older adults to use public transit systems, although physical limitations and the quality of public transport that is provided may limit their use of public transport. Policy measures that aimed at providing better transport infrastructure and services favouring public transit systems can address the problem of the quality of public transit to some extent; indeed, these policies have been implemented by many public authorities, as mentioned earlier. However, the financial burden and operational efficiency may prove to be key issues for the authority in providing sufficient and efficient public transit infrastructure and services for the elderly. It has been pointed out by some researchers that we may have entered a stage where it is necessary to explore solutions that allow new technologies to rationalise services, given that conventional public transport is unlikely to be able to efficiently cope with an ageing population, particularly those living in suburbs [26,54,65,66,67]. 

Wright et al. (2014) indicated that the rapid growth of funding demands for subsidised public bus services forced many local authorities to adopt flexible transport systems (FTS) as more affordable alternatives to heavily subsidised fixed-route bus services in the UK and Japan. In Japan, a Demand Responsive Transport service, as one of the two types of FTS, introduced by the Japanese government in 2006, allows the subsidy to be reduced by half (compared with the fixed-route buses replaced by the Demand Responsive Transport service) on some occasions. In the UK, the number of FTS schemes (delivered by 68 government authorities and 11 community transport organisations) increased from around 20 in 1997 to about 250 in 2011 [67]. Community transport schemes and subsidised taxis are also representative of transport policies for British seniors that were discussed by Metz (2003) [10]. In areas lacking local bus or rail services, community transport, a local passenger transport provision is often provided by community transport groups, voluntary organisations, and other non-statutory bodies. This type of non-profit transport provides door-to-door transport services. The services include: ‘community buses; group hire minibus services mainly providing trips of a social welfare nature; dial-a-ride services to meet the needs of mobility-impaired people; and voluntary social car schemes for a variety of medical, welfare, shopping and leisure purposes’ [10] (p. 378). Taxi concession schemes were also operated by some local authorities.

However, regulations could affect the provision of high-quality alternative transport services. Taking FTS as an example, in developed countries, formal FTS operates mostly according to predefined rules and regulations. Wright et al. (2014) summarised four principal forms of license and regulation—the type of service operated, type of operator, vehicle specification and drivers—in providing transport services to the public [67]. In contrast, the regulation of the FTS is still a challenge in some developing countries. For example, the absence of regulations regarding the planning and operation of FTS in India resulted in the provision of FTS being informal with regard to the route, timing, fare, and frequency of transport services. The informal provision of FTS led to problematic and inefficient operation, including long waiting and travel times, overcrowded travel environments, unsafe driving behaviour and poor integration with other transport modes [67].

However, regulation is double-edged. Despite the quality and efficiency achieved by FTS in the UK due to rules and regulations, it has been acknowledged that increased regulations could result in risks to the commercial viability of transport services. On the other hand, deregulation resulted in fixed-route bus services becoming concentrated on the high-demand, commercially viable routes in the UK and Japan; this left the fixed-route bus services in lower demand areas to regional and local governments to provide subsidised services. In comparison, in India, a lack of regulation of FTS allowed low entry and service standards for the service operators and thus provided affordable transport services to the poor [67]. 

Broome et al. (2012) investigated the replacement of a fixed-route bus service with a flexible-route bus service in Australia to see if this improves older people’s use of, and satisfaction with, flexible-route bus services. Hervey Bay in Queensland was selected to provide FTS service because of its ageing demographics (21.0 percent of its population aged 65 and over) and low population density (121.9 persons/km^2^) that allow FTS to be a reasonable alternative. The research revealed that both use, and useability of the bus service were significantly improved by doubling the number of users, while adding 30 percent of new users. Improvements in overall satisfaction were also seen in the aspects of operation times, information provision, bus characteristics (e.g., lower floor and handrails within the bus), driver friendliness, and signage about disability needs [26].

## 5. Transport Affordability

Reducing the travel cost of public transport for older people and thus encouraging their use of public transport to meet their mobility needs through subsidies for public transport is an approach that has been adopted by many public authorities in the world [68]. The UK is a good example of demonstrating the evolution of the concessionary travel policy for older adults. The policy evolves over years, with increasingly generous and consistent concessions provided in an increasingly extensive scale since early 1950s. According to Rye and Carreno (2008) and Mackett (2014), in England, older people have enjoyed a free trip by bus within their local authority areas since 2001. In Wales, concessions to older adults varied depending on the locations of their residences initially, but since 2002, they have enjoyed free travel by bus throughout Wales. In Scotland, older people enjoy free travel by bus after 9:30 a.m. on weekdays and all day on weekends. The concession to older adults varied depending on the locations of their residences since 2002. Since 2006, concessions for older people have been extended throughout Scotland [29,31]. The Concessionary Travel Pass (CTP) is very popular among older adults; according to data from the Department for Transport, UK, the proportion of eligible people holding it increased from 52 percent in 2002 to 79 percent in 2012 [69]. On the other hand, as suggested by Rye and Carreno (2008), the CTP policy was paid by the public sector through reimbursing bus operators for carrying concessionary passengers. A national formula is applied to calculate the amount of reimbursement. A considerable amount of money is spent on reimbursing bus operators, and an increasing number of older passengers means the increasing cost of concessionary fares to the public sector [31].

In terms of the outcomes of the CTP policy, research has indicated positive outcomes on travel behaviours, health and social inclusion. Mackett’s (2014) research confirmed that the introduction of the CTP policy has had the effect of increasing bus usage and access to services for older people, and for daily necessities there is a very significant use of the CTP [29]. Mackett also extracted evidence of more benefits for the social inclusion and the wellbeing of older people associated with the introduction of CTP from other studies, such as reduced travel costs to allow increased social activities, increased walking and physical activities contributing to better health, and enhanced access to services, facilities and social support improving mental health, and easing the transition from driving to ceasing driving. Research conducted by Jones et al. (2013) revealed that, from the perspective of older people, older people’s entitlement to free travel in London enhanced their sense of belonging to the city, increased the elderly’s feelings of social inclusion and wellbeing, and improved their sense of self-worth [28].

Although the policy of offering concessionary travel to older people has the effect of improving the usage of buses by older people, for those who live in areas that are not well-serviced by bus, a pass that offers free travel by bus does not help [29]. The proportion of eligible holders of the pass who live in rural areas is 66 percent, while the figure for the whole of the UK is 79 percent [69]. This raises the problem that massive subsidies have been spent on travel even though it only benefits for a proportion of the targeted population, questioning the effectiveness of the policy for decreasing social exclusion, as well as the equity of the distribution of subsidy [29]. In addition, Laverty and Millett (2015) also criticised the age for eligibility, which is due to be raised in the UK, with the effect of increasing the waiting time for older people to be eligible for free bus travel [69]. In Japan, the policy of older people’s discount fares or free passes for mass transit in some municipalities experienced similar problems. On the one hand, public transport revenue was reduced, increasing the financial burden on the municipalities. On the other hand, new public transport problems had arisen in both urban and suburban/rural areas. In urban areas, free and discounted passes increased the demand for public transport, thus decreasing the quality of public transport and forcing full-fare commuters back to private transport. In suburban/rural areas, there were complaints about the unequal distribution of free public transport benefits and requests for an extension of free public transit to suburban/rural areas [58].

Research has indicated that public transport fare concession schemes might need to consider different senior age groups (e.g., young-old, middle-old, and old-old). In Hong Kong, China, the government’s “Transport for All” policy highlighted the importance of creating accessible transport systems for old people. The elderly enjoy public transport concession ticket fares that cost only HK$2 per trip at any time. Elderly individuals who are eligible for public transport fare concession schemes are those aged 65 years and over. An increasing number of old people, who retired at the age of 60 in recent years, have become ineligible. It has been found that a scheme that provides subsidies for those aged 60 to 64 years by means of discounts would improve their mobility. In addition, considering the reduced mobility of those aged 80 years and over, completely free travel by public transport would encourage their participation in social activities. Moreover, schemes covering a wider range of transport modes in addition to railways, buses, ferries, and public light buses to allow more transport modes receiving government subsidies are also worth considering [68].

The economic objective of reducing subsidy requirements was one of the key motivations for the introduction of FTS and the replacement of conventional bus services. Although FTS is considered to be an alternative to fixed-route services that are significantly subsidised and underused, particularly in rural areas or when demand is low, considerable financial support for providing the services is still required. In the UK, fares only contribute to less than a quarter of the operating costs, and the balance is required to be fulfilled by public funds [67]. Coughlin (2009) highlighted a research project investigating the cost of fast-growing demand-response transit services in the US, viewed as part of a future public transport platform that provides as an alternative to driving. Surprisingly, of the overall cost of providing the service, ranging from $15 to over $50 per ride, the user only paid $2 [46]. In the UK, with regard to the affordability of community transport schemes and subsidised taxis, older people who have difficulties to use public transport are charged a fixed rate (for instance £1.50) for each journey, and the remaining fare, of up to an agreed maximum (for instance £10), is paid by the authority [10].

## 6. Transport Safety

The elderly constitute a vulnerable group of users of transport networks. The safety and mobility of the elderly, as a significant road safety issue should be prioritised in transport policy [70]. European countries (e.g., The Netherlands and Germany) have ensured that walking and cycling are safe and enjoyable alternatives to driving for the elderly. For example, traffic regulations in the two countries protect pedestrians and bicyclists. Even in cases where illegal movement by pedestrians or cyclists leads to accidents, the motorists would almost always at least partially be responsible for the accidents. If children or the elderly are involved in an accident, the motorist would usually be entirely responsible. In almost every case, the police and the courts hold that motorists should expect illegal or unsafe walking and cycling behaviour [71].

Safety is a key consideration of driver’s license possession policy for the elderly. Taking away driving licenses from unsafe older drivers would benefit not only the unsafe older drivers themselves but also road users as a whole. In Australia, road safety laws in all states and territories require drivers to report disabilities, health conditions and the effects of treatments that may impact their ability to drive safely [72]. Self-regulation of driving may be the preferred model for most older people [73]. However, studies have questioned the effectiveness of self-regulation of senior drivers as senior drivers reported little evidence of self-regulation [56]. In addition, there is a discrepancy between self-reported and objective measures [30]. A balanced transport policy needs to take into account the desire of older drivers to continue driving, as well as other safety, social and environmental concerns. Currently, the situation largely relies on older drivers’ continuing and responsible self-regulation [10]. It has been acknowledged that advancements in technology and motor vehicle design can help to improve the impact of impairments, but this is an area that still largely relies on the initiatives of private sectors such as car companies. Some research has indicated that the education of older drivers may be considered as a measure to enhance safe driving [58], particularly the education of high-risk older drivers to promote improved self-regulation [74].

Driver screening is a licensing policy that is implemented to impact on driver license possession and thus the travel behaviour of older adults. Driver screening requires older adults to provide proof of fitness to drive before license renewal and has been implemented in many countries. Siren and Haustein (2015) have shown that in 27 European Union countries, the licensing policies for older adults vary. Among the 27 countries, 21 have driver screenings for older adults. In most of the countries the age limit ranges from 50 to 70 years, although a few countries require drivers of all ages to provide proof of fitness to renew their licenses [52]. Normally, transport authorities gave general practitioners, ophthalmologists and psychologists greater responsibility in recognising unsafe drivers [12,52]. Despite this, vehicle accident data show that elderly drivers are less dangerous than young drivers [75,76]. There is a concern that it may not be equitable to revoke older people’s driving licensing while young drivers are more dangerous in their driving behaviour, and this question needs to be considered by policy-makers.

Undoubtedly, older people (including those with health problems) would prefer to maintain an active lifestyle and enjoy the benefits of active travel. To improve safety for pedestrians and cyclists, traffic regulations to slow car traffic speed have been adopted in many countries; it is only when vehicle speeds are low (e.g., 30 to 40 km/h) that pedestrians are safe (i.e., only rarely fatal damage and injury occur). General urban speed limits of 50 km/h and lower speeds in school zones and residential areas have been adopted by most OECD countries to accommodate the large numbers of pedestrians and cyclists. The ‘woonerf’ concept in residential areas, first developed in The Netherlands and common in European countries, is a successful example of traffic-calming [70]. Different transport modes share the transport space. Pedestrians and cyclists have priority, and traffic speed is limited to a very low level (e.g., 20 km/h). Older people’s attitudes towards the use of mobility scooters were examined in a study in Israel, indicating that mobility scooters were believed to have the effects of improving the mobility and quality of life of the elderly. However, a relatively low willingness to use mobility scooters was also reported, partly due to the lack of appropriate infrastructure for mobility scooters’ travel in the city. As a result, mobility scooters were perceived as a dangerous transport mode. Those aged 70–84, those who were single and those who had more outdoor daily activities were more willing to use mobility scooters. Mobility scooter users were careful in selecting travel routes and preferred slow traffic conditions to avoid traffic accidents [77]. With the adoption of new transport modes, e.g., scooters, e-scooters and e-bikes by the elderly for travelling, their safety aspects may need greater attention from policy-makers.

## 7. Discussion

The demographic change from a younger population to an older population, unlike many other unpredictable factors (e.g., economic development, technology advancement, and energy production), is generally foreseeable [46]. To better accommodate the mobility needs of an ageing population, a wide range of innovations in policies have been introduced, resulting in various outcomes with regard to older adults’ travel behaviour and health and social impacts. The key findings of this review are summarised and discussed below.

First, the review indicates that ageing is a process of change. The changes in older people’s living situations, housing, employment and interaction with others affect their transport and mobility needs [66]. Living in one’s own home or living in communal forms of habitation (see [43]), urban–rural migration (see [45]), maintenance of working, and physical and non-physical (supported by technology) interactions will pose challenges to decision-making on transport strategies for the elderly.

Second, it also can be seen from the review that the changing travel behaviour of the elderly, without a loss of welfare and misallocation of resources, is extremely difficult to achieve. For example, subsidised public bus services, in some cases, have significantly increased the financial burden for local authorities and created a series of problems related to social exclusion and the equity and efficiency of subsidy distribution [29,69]. Compared with subsidies for public transport, financial support for flexible-route bus transport may help to overcome the transport disadvantage that is common among those living outside metropolitan areas, if applied in an appropriate context [26]. However, such devising solutions that allow new technologies to rationalise services require a balanced regulation environment to be formulated to guarantee the provision of high-quality transport services.

Third, a considerable number of transport policies have been implemented in world cities using legislative and institutional approaches, that are aimed at addressing the availability, accessibility, affordability and safety of transport modes for older people. Based on the review, clear conclusions about the effectiveness of particular policies are difficult to make. The implemented policies had various outcomes with regard to older adults’ travel behaviour and health and social impacts in different areas in the world. It highlighted the requirement of future research to develop tools for assessing the effectiveness of innovative policies, to help in making decisions about the scale and balance of the effort and resources required. When developing evaluation tools to assess the effectiveness of innovative policies, a key consideration arising from this review is that a particular policy may have performance and outcomes, both positive and negative, with regard to financing, social inclusion, and the equity and efficiency of public resources, as well as individuals’ independence, wellbeing and quality of life. These aspects need to be comprehensively considered in evaluating the effectiveness of transport policies to allow for a balanced and unbiased assessment to support policy-making.

Fourth, transport innovations (e.g., technological solutions) may need to be considered by the authority in addressing the financial burden and operational efficiency issues in order to provide sufficient and efficient transport infrastructure and services for older people. Technology is playing an increasingly important role in changing every aspect of people’s lives, including the way we travel. Recent studies highlighted the importance and potential of transport innovations for the elderly [34], such as new private vehicle models [78,79], electric vehicles [80], shared mobility [81,82], and intelligent transport technology and support services [79,83] to help address transport challenges with regard to safety, equity, availability, accessibility and affordability. This would be a continuing research and policy agenda concerning transport provision for an ageing society.

## 8. Conclusions

This paper discussed policies for addressing the mobility needs of an ageing population. It highlighted areas in which legislative and institutional approaches can contribute to the increased mobility of the elderly. The paper’s findings have significant implications for planners and decision-makers, not only regarding the policy options for addressing the mobility needs of the elderly, but also for the challenges and outcomes a particular policy may have:

- Policies addressing older people’s mobility needs, using legislative and institutional approaches, should consider financing, social inclusion, and the equity and efficiency of public resources, as well as individuals’ independence, wellbeing and quality of life.
- Land use planning for housing provision for the elderly can contribute to optimising older people’s mobility needs, e.g., locating housing for older adults in close proximity to locations that the elderly visit frequently, such as shops, hospitals, and supermarkets. National, regional, and local authorities need to create legislative and institutional environments that facilitate close and collaborative work among planners, transport operators and health providers to ensure the services old people require are accessible, particularly for those living in rural areas and those without access to cars.
- A balanced transport policy needs to take into account the desire of older drivers to continue driving, as well as other safety, social and environmental concerns. Strong support is required to facilitate individual older drivers to stop driving at appropriate times and provide a transition to other transport options (particularly sustainable transport modes). The safety of the elderly as road users should be prioritised in transport policy. Traffic regulations need to protect senior pedestrians and bicyclists to make walking and cycling safe and enjoyable alternatives to driving for the elderly.
- Flexible public transport arrangements, integrated with new technologies, (e.g., FTS) may be more likely to cope efficiently with the physical limitations of an ageing population and rationalise services to achieve economic objectives. However, experiences in world cities have indicated the double-edged effects of regulation on such alternative transport services. On the one hand, increased regulation could allow for efficient operation while also risking the commercial viability of transport services. On the other hand, deregulation could allow affordable transport services but could also result in informal services that may lead to problematic and inefficient operation. A balanced regulation environment may need to be formulated for the provision of high-quality alternative transport services.
- The formulation of a concessionary travel policy needs to balance the following possible outcomes: first, potentially reduced public transport revenue may cause a heavy financial burden on municipalities; second, equity issues may arise due to the uneven provision of public transport services in different areas (e.g., urban and suburban/rural areas); and third, the advantages and disadvantages of including different senior age groups and different transport modes in the concessionary travel scheme. A balanced consideration of the depth and breadth of a concessionary travel policy applied to older people is needed to achieve the goals of efficient operation, affordability of public transport systems, and broad transport, health and social benefits.
- Alternative approaches (e.g., technology innovations) may need to be considered in addressing older people’s mobility needs in addition to the traditional infrastructure, service, legislative and institutional approaches to transport provision, particularly under the circumstances of insufficient funding support and inefficient transport systems.

This paper only focuses on the availability, accessibility, safety and affordability of transport in exploring policy areas, particularly legislative and institutional approaches to addressing the mobility needs of an ageing population. Other policy areas are not included in the paper and require further exploration in future research. Research on legislative and institutional approaches to addressing the mobility needs of an ageing population is very scarce. The purpose of this review is to map out existing knowledge on this topic, focusing on scholarly sources. In the future, with an increasing number of high-quality publications on this topic, an updated literature review, with further refined review and analysis approaches (e.g., a structured meta-analysis or a systematic quantitative literature review), would significantly contribute to the advancement of knowledge in this field. In addition, since ageing experiences and related policies are far from homogenous, it would be valuable in future research to examine the similarities and differences in policy in different geographical areas/nations. Moreover, most of the experiences with regard to transport provision for an ageing society via legislative and institutional approaches have been documented in developed countries. With an increasingly rapid demographic transition from a younger population to an older population in developing countries, addressing the mobility needs of an ageing society presents a major challenge in developing future transport policy in developing economies. Empirical evidence is needed regarding transport provision for the ageing society via legislative and institutional approaches in developing countries.

A key issue that became apparent in the provision of transport infrastructure and service is that future transport policies for addressing older adults’ mobility needs require the consideration of transport desired by older adults, than exclusively focusing on what they need. When providing legislative and institutional support for older adults’ mobility, it is vital to realise that the objective of transport alternatives such as transit is not to simply move older adults from origin to destination, but to provide a desirable transport system that is affordable, efficient, convenient, comfortable and enjoyable, and fully accommodates older adults’ travel characteristics and concerns. Accelerating the development of such transport infrastructure and services that are financially acceptable, should be a worldwide imperative.

## Figures and Tables

**Table 1:** Review protocol.

Item	Criteria
Review questions	What policies have been implemented for addressing the mobility needs of ageing populations, particularly with regard to legislative and institutional options; how have these policies been implemented; what were the outcomes and issues?
Inclusion criteria	Studies on transport provision for an ageing society from a policy perspective (particularly related to the legislative and institutional approaches)
Exclusion criteria	Non-English publications; papers that do not discuss transport policies; papers that do not discuss transport for the elderly
Literature search	Sources: databases (Google Scholar and ScienceDirect)
	Search terms: ‘transport policy’, ‘housing’, ‘affordability’, ‘availability’, ‘accessibility’, ‘safety’, ‘travel behaviour’, ‘mobility’, ‘regulation’, ‘subsidy’, ‘public transport’, ‘driver license’, ‘active transport’, ‘the elderly’, ‘older people’ ‘senior’, and ‘ageing’; search title and abstract with no limitation on publication year
